# Identification of Multiple Genetic Loci Related to Low-Temperature Tolerance during Germination in Maize (*Zea maize* L.) through a Genome-Wide Association Study

**DOI:** 10.3390/cimb45120602

**Published:** 2023-11-29

**Authors:** Tao Yu, Jianguo Zhang, Jingsheng Cao, Shujun Li, Quan Cai, Xin Li, Sinan Li, Yunlong Li, Changan He, Xuena Ma

**Affiliations:** 1 Maize Research Institute of Heilongjiang Academy of Agricultural Sciences, Harbin 150086, China; caoj.s@163.com (J.C.); cq6539@163.com (Q.C.); maize_lee@163.com (X.L.); xuena19920207@163.com (X.M.); 2 Key Laboratory of Biology and Genetics Improvement of Maize in Northern Northeast Region, Ministry of Agriculture and Rural Affairs, Harbin 150086, China; 3 Key Laboratory of Germplasm Resources Creation and Utilization of Maize, Harbin 150086, China; 4 Keshan Branch of Heilongjiang Academy of Agricultural Sciences, Qiqihaer 161000, China

**Keywords:** abiotic stress, QTLs, low-temperature tolerance, genome-wide association study (GWAS)

## Abstract

Low-temperature stress during the germination stage is an important abiotic stress that affects the growth and development of northern spring maize and seriously restricts maize yield and quality. Although some quantitative trait locis (QTLs) related to low-temperature tolerance in maize have been detected, only a few can be commonly detected, and the QTL intervals are large, indicating that low-temperature tolerance is a complex trait that requires more in-depth research. In this study, 296 excellent inbred lines from domestic and foreign origins (America and Europe) were used as the study materials, and a low-coverage resequencing method was employed for genome sequencing. Five phenotypic traits related to low-temperature tolerance were used to assess the genetic diversity of maize through a genome-wide association study (GWAS). A total of 14 SNPs significantly associated with low-temperature tolerance were detected (−log10(P) > 4), and an SNP consistently linked to low-temperature tolerance in the field and indoors during germination was utilized as a marker. This SNP, 14,070, was located on chromosome 5 at position 2,205,723, which explained 4.84–9.68% of the phenotypic variation. The aim of this study was to enrich the genetic theory of low-temperature tolerance in maize and provide support for the innovation of low-temperature tolerance resources and the breeding of new varieties.

## 1. Introduction

The northern spring corn area is an important corn production area and commercial grain base in China, located at the northern end of China’s golden corn belt. However, due to the special geographical location and environmental conditions, low temperatures in spring are an important source of non-biotic stress that affects the seedling quality in this area, which seriously restricts the yield and quality of the corn produced. The low-temperature tolerance of maize belongs to a quantitative trait controlled by multiple genes. In recent years, with the development of molecular biology, scholars have carried out quantitative trait locis (QTL) analyses on its low-temperature tolerance, locating maize’s low-temperature tolerance on chromosomes 1–10. One QTL was located in an interval on chromosome 6, which was associated with three low-temperature tolerance traits and could explain 18.1–32.8% of the phenotypic variation [1]; twenty-six QTLs, associated with seed vigor, were detected under low temperatures during maize’s germination stage on chromosomes 2, 3, 5, and 9, alongside five meta-QTLs [2]. In the 176 IBM Syn10 doubled-haploid population from the B73 × Mo17 cross, there were thirteen QTLs associated with a low-temperature germination ability, three B73 upregulated genes, and five Mo17 upregulated genes found by combining the RNA-Seq technology and QTL analysis [3]. A recombinant inbred line population (IBM Syn4 RIL) from a B73 and Mo17 cross was used to identify QTLs and investigate the genetic architecture under low-temperature conditions at a young seedling stage: two QTLs (bin 1.02 and bin 5.05) with a high additive impact were detected, which were associated with cold tolerance [4]. A total of 406 recombinant inbred lines from a multi-parent, advanced-generation, intercross population were used and, as a result, many cold tolerance-related traits were recorded: the 858 SNPs were found that were significantly associated with all traits, which indicated that most QTLs are related to chlorophyll and Fv/Fm; the authors also located most of the QTLs in specific regions, particularly bin 10.04 [5]. An F_2_ population was constructed from the cross of IB030 and Mo17 to map QTLs associated with cold tolerance via QTL-seq and transcriptomic integrative analyses, and two positively regulated genes (*ZmbZIP113* and *ZmTSAH1*) that control the low-temperature germination ability were identified [6]. Scholars performed QTL mapping on an IBM (intermated B73 × Mo17) Syn10 doubled-haploid (DH) population, and twenty-eight QTLs that significantly correlated with low-temperature germination were detected, and these QTLs explained 5.4–13.34% of the phenotypic variation. In addition, six QTL clusters were produced by fourteen overlapping QTLs on every chromosome, except for chromosomes 8 and 10 [7]. The identification of molecular marker loci associated with QTLs or genes can contribute to the study of the cold-tolerance mechanism of maize and could be further used for breeding cold-tolerant inbred lines or hybrids. The QTLs controlling low-temperature tolerance during the germination stage are distributed on chromosomes 1–10, and there are few QTLs that have been consistently identified using different methods and materials, with large intervals. At the same time, there is more than one main QTL interval, so it is necessary to continue to mine consistent, main QTLs and identify candidate genes.

In recent years, the construction of reference genomes, such as B73, MO17, W22, PH207, and CML247, has enabled the widespread application of high-throughput single-nucleotide sequence markers, greatly improving the accuracy and depth of maize’s whole genome sequencing and marker development. Relying on the progress of whole-genome sequencing technology and the development of whole-genome association analysis models and methods, and due to the higher level of genetic diversity in the mapping populations, GWAS has been used to analyze the variations of maize seedling and germination traits under low-temperature conditions. This is because GWAS offers increased mapping resolution and accuracy. A total of 338 cross experiments showed that some QTLs for four seedling cold-tolerance traits were detected using GWAS; thirty-two significant loci and thirty-six candidate genes related to stress tolerance were identified, suggesting that heterosis may be related to maize’s cold tolerance [8]. To identify and analyze cold-tolerance traits in 306 dent inbred lines and 206 European flint inbred lines from temperate regions, indirect cold-tolerance traits such as days from sowing to germination, relative chlorophyll content, and quantum yield of photosystem II were studied. Using the GWAS technology, 49,585 SNPs were used for genotyping, and associations between SNPs and cold-tolerance genes were located in both types. A total of 275 significant associated markers were found, and some candidate genes were consistent with current studies and previous reports [9]. A GWAS of 125 maize inbred lines was studied using 10 low-temperature tolerance traits during the seedling stage and the germination stage; finally, 43 SNPs were identified as being associated with low-temperature tolerance [10]. A study conducted a GWAS on 375 inbred lines grown outdoors and in an artificial climate chamber and identified 19 markers associated with low-temperature tolerance. These markers explained 5.7% to 52.5% of the phenotypic variation in the chlorophyll fluorescence parameters during the seedling stage. The candidate genes identified near the markers were related to ethylene signaling, brassinosteroid, and lignin synthesis [11]. A study employed two cold-tolerant inbred lines, 220 and P9-10, and two susceptible lines, Y1518 and PH4CV, to generate three F_2:3_ populations to detect QTLs associated with the low-temperature germination ability of seeds. Forty-three QTLs were detected, explaining 0.62% to 39.44% of the phenotypic variation. Among them, 17 QTLs explained more than 10% of the phenotypic variation, with 16 inheriting the favorable alleles from the tolerant lines. After constructing a linkage map, three meta-QTLs were identified, including at least two initial QTLs from different populations. mQTL1-1 includes seven initial QTLs for germination and seedling traits, with three explaining more than 30% of the phenotypic variation [12]. GWAS was used to conduct a germination test on 282 inbred lines and 17 loci associated with cold tolerance were identified [13]. GWAS and QTL mapping were performed on two populations; a total of four associated SNPs and twelve QTLs related to cold tolerance were identified, and the results showed that the *Zm00001d002729* gene was a potential factor, with its overexpression being able to improve the cold tolerance of crops [14]. Using GWAS, a total of 30 SNPs were identified that were related to low-temperature tolerance during seed germination, and fourteen candidate genes were found to be directly related to these SNPs; in a further study of the linkage between these candidate genes and low-temperature tolerance, ten differentially expressed genes were identified via RNA-seq analysis [15]. Fifteen significant SNPs related to seed germination were identified via GWAS under cold stress in 300 inbred lines; among them, three genomic loci were repeatedly associated with multiple traits. In further candidate gene association analysis, *Zm00001d010458*, *Zm00001d050021*, *Zm00001d010454*, and *Zm00001d010459* were identified as cold-tolerance germination-related candidate genes [16]. A total of 187 significant SNPs were identified via GWAS in 836 maize inbred lines, and there were 159 QTLs for emergence and traits related to early growth [17]. Many of the QTL and GWAS analyses have been widely used to express large variations in cold tolerance of maize, and these cited results open up new possibilities for improving cold tolerance and understanding the molecular and genetic mechanism of cold tolerance in maize. In addition, QTL mapping and GWAS can be applied as resources for conducting marker-assisted selection of cold-tolerant varieties, and we can use genomic selection technology to predict cold-tolerant varieties in large maize populations [18].

In this study, a population of 296 excellent inbred lines of maize from China and abroad was used as the study material, and their genotypes were analyzed via genome resequencing. The germination stage was then subjected to low-temperature tolerance identification in the field and laboratory, and indicators such as germination rate and germination index were detected. The TASSEL 5.0 method was used for GWAS to identify associated SNPs, aiming to provide theoretical support and material resources for the gene mining and breeding of low-temperature tolerance in maize.

## 2. Materials and Methods

### 2.1. Plant Materials

We selected 296 representative inbred lines of maize (*Zea maize* L.) from both domestic and international sources, including 232 domestic lines, 36 US lines, and 28 European lines (see Appendix A). The seeds were provided by the maize research institute of Heilongjiang Academy of Agricultural Sciences, and the seed germination rate was above 95%.

### 2.2. Identification of Low-Temperature Tolerance during Germination in the Field 

The experiment was conducted in the period of 2017–2019 at the experimental field of Heilongjiang Academy of Agricultural Sciences. The soil in the field was calcic soil, which is neutral, flat, and uniform. The experiment was conducted in two stages. In the first stage, seeds were sown as soon as the soil temperature at 5–10 cm depth reached and remained above 5 °C, while in the second stage, seeds were sown when the soil temperature at 5–10 cm depth remained stable at or above 10 °C. After sowing, timely irrigation was carried out. A randomized block design with two rows, each 5 m in length, with 20 cm between plants and 65 cm between rows, was employed with single-seed sowing, and three replicates were used. Daily records of soil temperature, maximum and minimum temperatures in the field, and daily average temperature were noted during the experiment. Natural low-temperature treatment was applied to the seeded plots, and the number of seedlings that germinated was recorded accurately every day. After the cessation of seedling germination, the field seedling germination rate was calculated, and the relative seedling germination rate and relative seedling germination index were determined as follows:

germination rate (%) = (number of germination seeds/total number of seeds) × 100

relative germination rate (%) = (germination rate of early sowing treatment/germination rate of appropriate sowing treatment) × 100

germination index = ∑Gt/Dt (Gt represents the number of germination seeds at time t, and Dt represents the corresponding days)

relative germination index (%) = (germination index of early sowing treatment/germination index of appropriate sowing treatment) × 100

### 2.3. Identification of Low-Temperature Tolerance during Germination in the Laboratory 

Fifty plump seeds of each inbred line were selected, surface-sterilized with 0.5% sodium hypochlorite solution for 5 min, and then rinsed three times with sterile water. The sterilized seeds were transferred onto a culture dish lined with filter paper and covered with 3 cm thick vermiculite that was kept moist; these seeds were then allowed to germinate in a low-temperature incubator under dark conditions. Two low-temperature treatment stages were set up; the first included germination at 5 °C for 7 days, followed by 15 °C for 7 days and then 25 °C for another 7 days, whereas the control was germinated at 25 °C for 21 days. The germination of seedlings with germ breaking through the vermiculite was defined as germination, and the number of emerged seedlings was recorded daily. The experiment was carried out in three replicates. The germination rate was calculated, and the relative germination rate and relative germination index were determined to be 2.2. 

### 2.4. Phenotypic Analysis 

Data organization and analysis were performed using Microsoft Office Excel 2016 and R version 3.6.2 [19]. Basic statistical quantities were calculated using Microsoft Office Excel. ANOVA was performed using the aov function in the R language with a random blocking model [20]. Correlation analysis was conducted using the cor function in the R language.

### 2.5. Analysis of Genotype

#### 2.5.1. Analysis of SNPs

The modified CTAB method [21] was used to extract the genomic DNA of 296 maize inbred lines, DNA quality was detected using a NanoDrop 2000 spectrophotometer (Thermo Fisher Scientific Inc., Kanagawa, Japan) and 0.80% agarose gel electrophoresis, and qualified DNA samples were used for SNP typing. In this study, the genotype of the association analysis population was analyzed by means of genotyping by sequencing (GBS). A combination of MseI, NlaIII, and EcoRI endonucleases was used to cleave the genomic DNA of the maize inbred lines, ligate the linker, construct the library, and sequence. After obtaining the original resequencing results, mutation detection was carried out using GATK (Genome Analysis Toolkit). Clean reads were compared to the reference sequence RefGen_v4 B73 using Bowtie2, and the resulting sam file was labeled, sorted, and removed through Picard to obtain a bam file for GATK. The indel around the bam file was re-aligned using GATK, and then SNP/INDEL analysis was performed using GATK’s HaplotyeCaller command. After merging all obtained vcf files, the SNP genotype data of the 296 maize inbred lines were finally obtained.

#### 2.5.2. Analysis of Population Structure

Population structure analysis was performed using the LEA software package v3.3.2 in R [22]. First, the TASSEL 5.0 software [23] was used to remove SNP markers with rare allele frequencies (minor allele frequencies, MAFs) of less than 0.05, and the remaining SNP markers were exported in the ped format. The ped files were converted to geno- and lfmm-formatted genotyping data using the ped2geno and ped2lfmm functions of the LEA software package v3.3.2. Then, the snmf function of the software package was used to calculate the population structure. The number of subpopulations was set from 1 to 10, and each subpopulation was repeated 10 times. The cross-entropy criterion for subpopulation allocation was calculated using the cross-validation method built into the snmf function, and the appropriate number of subpopulations was selected based on this criterion. The Q-matrix was determined based on the maximum genetic similarity of each inbred line.

#### 2.5.3. Analysis of LD 

In the analysis of linkage disequilibrium (LD), the TASSEL 5.0 software [23] was first used to remove SNP markers with a minor allele frequency (MAF) of less than 0.05, and the markers were divided into 10 categories according to the 10 chromosomes and arranged based on their physical position from smallest to largest using B73 RefGen_v4 as a reference. Then, a sliding window approach was used to calculate the LD between these SNP markers, with each window consisting of 100 SNPs and sliding by 1 SNP at a time. The LD between markers was measured using *r*^2^ [24]. After obtaining the LD between pairs of SNP markers, an LD decay plot was generated as a function of the physical distance between the markers.

### 2.6. Analysis of Genome-Wide Association

Genome-wide association analysis was mainly performed using TASSEL 5.0 [23]. Based on the analysis of 296 maize inbred lines, high-quality SNP markers were selected for subsequent analysis by removing SNP markers with minor allele frequencies of less than 0.05 using the TASSEL software [23]. The kinship matrix was calculated using the TASSEL software to estimate the relatedness among the 296 maize inbred lines. The first 10 principal components were calculated using the TASSEL software’s PCA function as population structure parameters. The low-temperature tolerance indices of the 296 domestic and foreign elite maize inbred lines were used, together with SNP genotypes, population structures, and relatedness, to perform genome-wide association analysis using a mixed linear model in the TASSEL software. False positives resulting from multiple comparisons in the genome-wide association analysis results were controlled using the Benjamini and Hochberg method for controlling the false discovery rate, and the false discovery rate was set to 0.10 [25].

## 3. Results

### 3.1. Phenotypic Analysis of Low-Temperature Tolerance during Germination in the Field

A variance analysis was performed based on the relative germination index and field relative germination rate of 296 maize inbred lines (Table 1). The results show that there were highly significant differences in genotype, environment, and the interaction between genotype and environment for the relative germination index, with all results reaching a significance level of 0.001. For the field germination rate, there were also highly significant differences in genotype and environment, and both reached a significance level of 0.001. Overall, the relative germination index and field germination rate indicate significant differences in low-temperature tolerance among different maize inbred lines.

The phenotypic analysis results of relative germination rate and relative germination index of the 296 inbred lines under natural field conditions are shown in Table 2. The minimum, maximum, and average values of the field average germination rate for the inbred lines are 28.00%, 100.00%, and 66.62%, respectively. The number of inbred lines that fall on the right side of the mean is higher than those on the left side of the mean. In comparison, the relative seedling germination index in 2017 was similar to that in 2018, which was 86.80% and 83.15%, respectively. Overall, the distribution of the field-averaged relative seedling germination rate, the 2017 relative seedling germination index, and the 2018 relative seedling germination index vary widely, and the low-temperature tolerance variation in the inbred lines is relatively high, with a generally normal distribution.

Under suitable sowing and early sowing conditions, the average seedling germination rate of the 296 inbred lines was 86.87% and 62.48%, respectively. Low-temperature stress significantly reduced the germination rate of each inbred line. There were significant differences among the 296 inbred lines in their relative germination index, which could reduce genotypic differences among the inbred lines and better reflect the differences in their cold tolerance. 

### 3.2. Phenotypic Analysis of Low-Temperature Tolerance during Germination in the Laboratory

The results of the variance analysis of indoor relative germination rate are shown in Table 3. The differences between genotypes, environments, and the interaction between genotype and environment were highly significant, reaching a significance level of 0.001. The differences between genotypes and blocks were also highly significant, reaching a significance level of 0.001. Overall, the indoor germination rate indicates significant differences in cold tolerance among different inbred lines.

The phenotypic analysis results of the indoor relative germination rate of the inbred lines are shown in Table 4. From the table, it can be seen that the average relative germination rates in 2018 and 2019 were 79.51% and 84.60%, respectively. Overall, the distribution range of the indoor relative germination rates in 2018 and 2019 was relatively large, indicating a high variation in cold tolerance among different inbred lines.

### 3.3. Correlation Analysis of Low-Temperature Tolerance during Germination between Field and Indoor

The correlation analysis showed that the indoor relative germination rate in 2018 was significantly correlated with the indoor relative germination rate in 2019 and the relative germination rate in the field, with correlation coefficients of 0.67 and 0.18, respectively, and both reached a significant level of 0.001 (Table 5). The indoor relative germination rate in 2019 was significantly correlated with the relative germination rate in the field, with a correlation coefficient of 0.20, which reached a significant level of 0.001. The relative germination rate in the field was significantly correlated with the field relative germination indices in 2017 and 2018, with correlation coefficients of 0.50 and 0.49, respectively, and both reached a significant level of 0.001. Among the significantly correlated indicators, the correlation coefficient between the indoor relative germination rate in 2018 and that in 2019 was the highest, reaching 0.67, while the correlation coefficient between the indoor relative germination rate in 2018 and the relative germination rate in the field was the lowest, at 0.18.

### 3.4. Analysis of Genotype 

#### 3.4.1. Analysis of SNPs

A total of 24,042 high-quality SNP markers were identified across the entire maize genome (Table 6). The identified SNPs were distributed relatively evenly across the ten chromosomes, with the highest number found on chromosome 1 (3687 SNPs), followed by chromosome 2 (3217 SNPs). The lowest number of SNPs was found on chromosome 10 (2976 SNPs). Of the 24,042 SNPs identified, 98% had a minor allele frequency greater than 0.05, and 36% had a minor allele frequency greater than 0.1. Additionally, SNPs with different minor allele frequencies were distributed relatively evenly across the ten chromosomes.

#### 3.4.2. Analysis of Population Structure 

**Figure 1:**
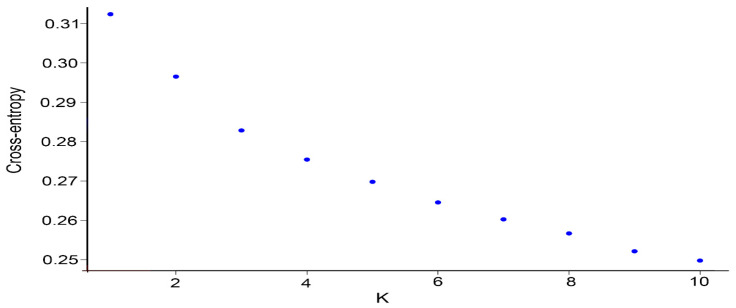
Variation in the clustering standard cross-entropy as the number of subgroups K increases.

**Figure 2:**
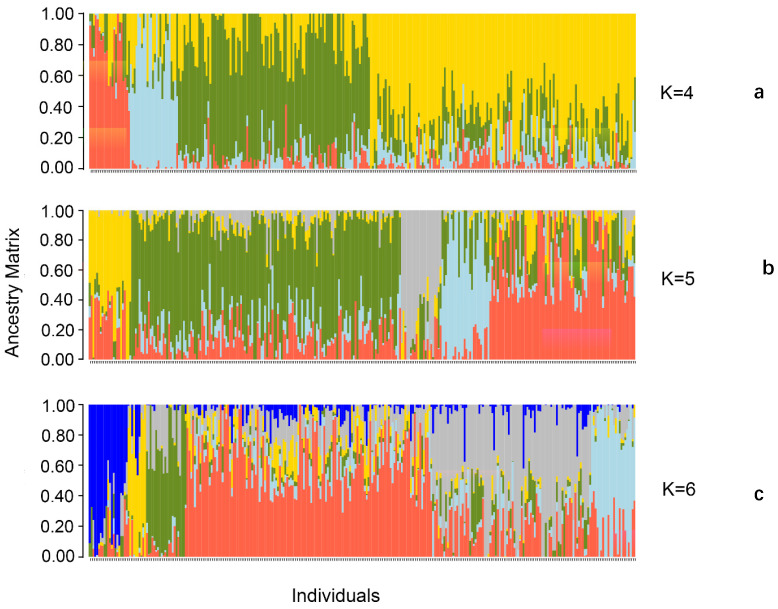
The population structure of subgroups from K = 4 to K = 6. Note: (**a**) K = 4; (**b**) K = 5; and (**c**) K = 6.

We used the snmf function with a cross-validation technique to calculate and select the appropriate number of subpopulations based on the standard, and we classified the individuals based on their maximum genetic similarity (Q-matrix). When the number of subpopulations is set from 1 to 10, the cross-entropy criterion for assigning subpopulations gradually decreases, but no obvious turning point is observed (Figure 1). When the number of subpopulations is varied, the clustering of the inbred lines is clearly distinguished (Figure 2). When the number of subpopulations is set to five, the 296 excellent inbred lines are divided into five subpopulations. Subpopulations A, B, C, D, and E include 21, 22, 178, 10, and 65 inbred lines, respectively.

#### 3.4.3. Analysis of LD

**Figure 3:**
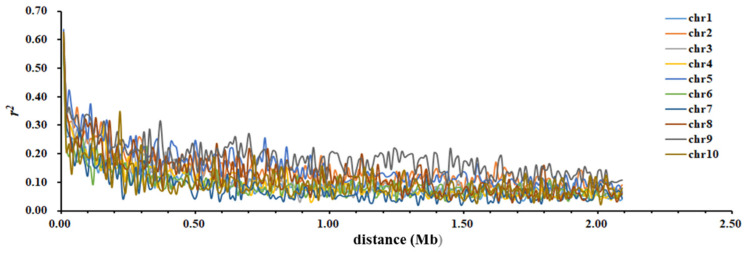
The attenuation in maize chromosome LD with an increase in SNP distance.

Using 23,497 SNP markers with an MAF greater than 0.05 for LD analysis, the LD *r*^2^ between these SNP markers basically decreases with an increase in genetic distance between the markers, and all values are distributed between 0.000 and 1.000 (Table 7, Figure 3). The average value of LD between the SNP markers on chromosome 9 is the highest, at 0.122, while the average value on chromosome 7 is the lowest, at 0.052 (Table 7).

### 3.5. Analysis of Genome-Wide Association 

#### 3.5.1. Genome-Wide Association Analysis of Low-Temperature Tolerance in the Field 

**Figure 4:**
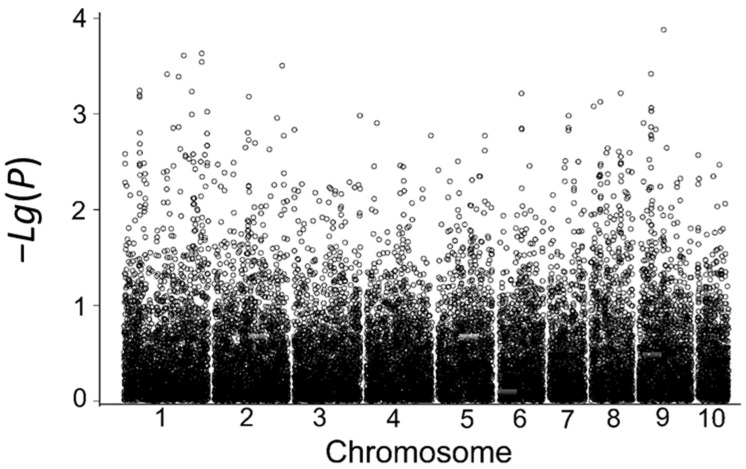
Correlation analysis based on the relative germination index in 2018.

Using the MLM model in the TASSEL software at a significance threshold of *p* < 1 × 10^−4^, seven SNP markers associated with low-temperature tolerance were detected based on the average relative germination rate and the relative germination indices in the field in 2017 and 2018. These markers are located on chromosomes 5, 6, 7, and 10, and their phenotypic contributions range from 5.03% to 9.68% (Table 8, Figure 4). Among them, five significantly associated SNP markers were identified based on the relative germination index in 2018, including marker.17002, marker.17003, marker.17009, and marker.17105 located on chromosome 6 and marker.19874 located on chromosome 7, which explained 6.55%, 6.55%, 5.86%, 8.46%, and 7.30% of the phenotypic variation, respectively. No associated SNP loci were identified for the year 2017, which might be due to the lack of effective low-temperature stress between early sowing and sowing at the optimum time in that year.

#### 3.5.2. Genome-Wide Association Analysis of Low-Temperature Tolerance in the Laboratory

**Figure 5:**
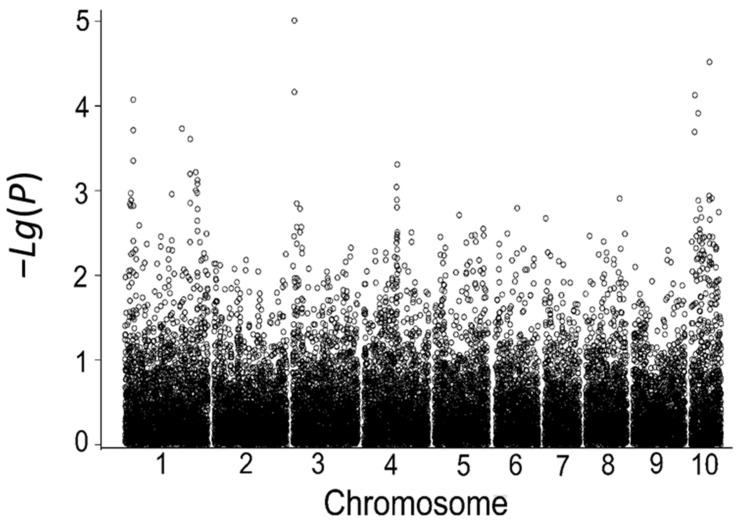
Correlation analysis based on the indoor relative germination index in 2018.

**Figure 6:**
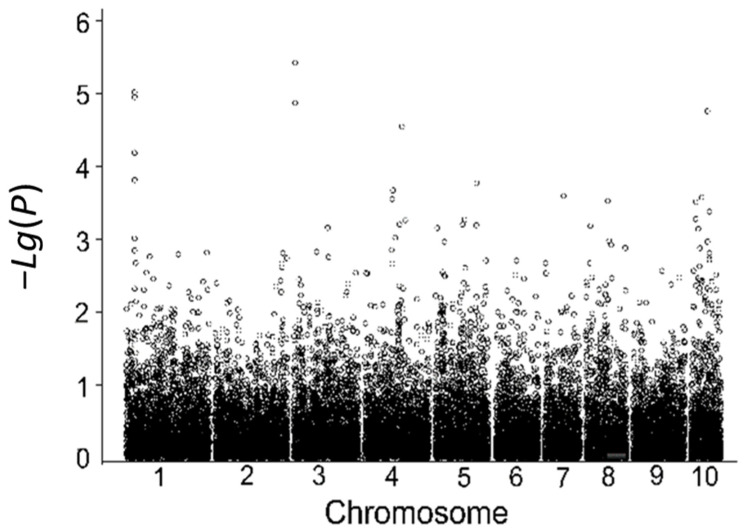
Correlation analysis based on the indoor relative germination index in 2019.

Using the relative germination rate in the laboratory during the germination stage as the indicator, a total of 14 SNP loci associated with low-temperature tolerance during germination were detected; these SNP markers are located on chromosomes 1, 3, 4, 5, and 10, explaining 4.84% to 9.68% of the phenotypic variance. Among them, six significantly associated SNP markers were identified using the relative germination rate in 2018 (Table 9, Figure 5), and eight significantly associated SNP markers were identified using the relative germination rate in 2019 (Table 9, Figure 6).

#### 3.5.3. Consistency Analysis of SNP Markers Associated with Low-Temperature Tolerance 

Eight significantly associated SNP markers were identified using the indoor relative germination rate in 2019, mainly distributed on chromosomes 1, 3, 4, and 10. When using the indoor relative germination rate in 2018 and the field relative germination index in 2018, five significantly associated SNP markers were identified for each. No significantly associated SNP markers were identified based on the germination index in 2017. Overall, significantly associated SNP markers were distributed on chromosomes 1, 3, 4, 5, 6, 7, and 10, with most markers on chromosome 1 (up to nine), and no significantly associated SNP markers were found on chromosomes 2, 8, and 9. The −*Lg*(*p*) values of significantly associated SNP markers ranged from 4.00 to 6.86, with an average of 4.65. The phenotypic variation explained by a single SNP marker ranged from 4.84% to 9.68%, with an average of 6.13%. 

Significantly associated SNP markers also showed clustered distribution. Using the indoor relative germination rates in 2018 and 2019, four significantly associated SNP markers (marker.1723, marker.1724, marker.1726, and marker.1729) were identified in the interval of 31,809,859–31,954,983 on chromosome 1, with an average distance of 36.28 Kb between markers. Using the indoor relative germination rates in 2018 and 2019, a significantly associated SNP marker (marker.8339) was identified on chromosome 3 at position 6,292,001, explaining up to 5.61% of the phenotypic variation. Using the indoor relative germination rate in 2018 and the field relative germination rate in 2019, a significantly associated SNP marker (marker.8340) was identified on chromosome 3 at position 6,292,053, explaining up to 6.87% of the phenotypic variation. Using the indoor relative germination rate in 2019 and the field relative germination rate in 2019, a significantly associated SNP marker (marker.14070) was identified on chromosome 5 at position 2,205,723, explaining up to 9.68% of the phenotypic variation. 

## 4. Discussion

In recent years, with the rapid development of sequencing technology and statistical algorithms, GWAS has become one of the most effective methods for identifying genetic variants associated with important agronomic traits in crops [26,27,28,29]. Compared to the traditional linkage analysis, GWAS can use natural populations as materials directly; it can detect more QTLs than traditional QTL mapping by using biparental populations because it uses a larger number of molecular markers and datasets from hundreds of maize inbred lines, which have a rich allelic diversity [30,31]. Moreover, GWAS can analyze multiple phenotypic traits in multiple environments and across multiple time points at the same time. Its high-throughput sequencing and high precision have greatly improved the efficiency of crop breeding [32,33]. Currently, GWAS has greatly advanced genetic research on maize functional genomics [34]; many agronomic traits such as flowering time, leaf angle, leaf size, and disease resistance have been identified in maize. For example, using 368 maize inbred lines and approximately 1 million SNPs, a GWAS analysis successfully detected 74 loci associated with seed oil content and fatty acid composition in maize [35,36]. The US-NAM population was used to detect maize flowering time variants, and a total of 90 flowering time regions were identified in the whole genome via GWAS; among them, one third of regions were associated with the environmental sensitivity of maize flowering time [37]. In another study, 513 inbred lines were used to identify 678 SNPs associated with 17 agronomic traits via GWAS, such as plant height, seed morphology, and flowering time; the results found that 54.3% of these SNPs were associated with at least two or more agronomic traits [38]. A total of 217 inbred lines were genotyped using the GBS technology, and 39 SNPs were identified to be significantly associated with fumonisin resistance in maize kernels based on GWAS analysis [39]. A panel of 143 elite lines were genotyped by using the MaizeSNP50 chip, combined with GWAS and transcriptome analysis; the results showed that 15 common quantitative trait nucleotides were associated with maize white spot, and SYN10137-PZA00131.14 was identified as a key genetic region for improving resistance to MWS; in this region, three candidate genes were identified [40].

Maize can grow in cool-temperate climates but is often exposed to cold temperatures in spring, which can affect seedling growth. Currently, although studies have shown that the growth and development of maize plants are closely related to low temperatures, the genetics of low-temperature tolerance in maize is not well understood. For example, low-temperature stress can increase the expression of related genes, resulting in the accumulation of folate in maize plants [41]. Cold stress can result in a series of physiological responses, such as the expression of osmotic stress-related genes, accumulation of ROS, activities of antioxidant enzymes, and levels of plant hormones and MDA production [42,43,44,45]; thus, plants need to stabilize cell membranes and biologically active proteins in order to survive under low-temperature conditions. However, low-temperature tolerance in maize is a complex trait because the identification and evaluation of low-temperature tolerance traits are complex and have not been standardized. Classic quantitative genetics studies have shown that low-temperature tolerance is controlled by multiple genes and is easily affected by environmental conditions. Quantitative genetic analyses of cold tolerance have shown that genotype, additive effects, growth stage, heterosis, and reciprocal and environmental factors are all involved in the expression of cold tolerance in maize [46]. Six maize lines were used to evaluate the expression of CAT, APX, SOD, and other genes; the results showed that there was heterosis for germination under cold stress, and non-additive genes were more important [47]. The studies cited above show that the genetics mechanisms of low-temperature tolerance in maize are very complicated.

Maize has rich genetic variability, a fast LD decay rate, and abundant information on SNP loci, so maize is an ideal model crop for GWAS analysis [48]. In this study, we used GWAS to identify the genetic loci associated with five traits related to low-temperature tolerance during germination. We identified 30 markers significantly associated with low-temperature tolerance, which were located on chromosomes 1, 2, 3, 4, 5, 6, 7, and 10. Two markers (marker.17002 and marker.17009) significantly associated with the relative germination index in the field in 2018 were located in bin 2.05. This interval has been mapped to several traits under different temperature conditions, including SPAD values [49], antioxidant activity under cold treatment, chlorophyll b, chlorophyll a + b, and Fv/Fm under different temperatures and sowing times [50]. Marker.7569, significantly associated with the relative germination index in the field in 2018, was located in bin 2.06 and was involved in the photosynthetic traits of the third leaf under 15 °C conditions, including CO_2_ assimilation rate and ΦPSII [1]. Marker.19874, located in bin 2.08, was associated with hundred-grain weight under 14 °C/10 °C (day/night) conditions [51]. Some of these associated markers are consistent with previous studies on low-temperature tolerance, although the traits they are associated with may differ, which may be due to pleiotropy.

Eight significant SNPs related to relative germination rate were detected using the indoor relative germination rate in 2019, and five significant SNPs related to low-temperature tolerance were detected using both the indoor relative germination rate in 2018 and the field relative germination index in 2018. Overall, these significant SNPs related to low-temperature tolerance were distributed on chromosomes 1, 3, 4, 5, 6, 7, and 10, with most SNPs distributed on chromosome 1 (nine SNPs). Previous studies have also shown that SNPs that are associated with seedling-related traits in maize under cold stress are concentrated on chromosomes 1, 2, 3, 5, 6, 8, and 10 [13,52,53]. The above research results further indicate that the cold tolerance of maize is a polygenic quantitative trait controlled by multiple genes. Using polygenic aggregation or multiple molecular markers for the genetic improvement of cold tolerance in maize is an effective strategy. No significant SNPs related to relative germination index were detected in 2017 using GWAS analysis, indicating that low-temperature tolerance in maize may be easily affected by environmental conditions, particularly climate conditions during the growing season. Some scholars have reported that the gene expression related to cold tolerance was affected by the environment of maize [54]. Under controlled conditions, the highest number of significant SNPs related to relative germination rate was detected using the indoor relative germination rate in 2019 (nine SNPs), further demonstrating that controlling low-temperature environmental conditions is important for identifying variations in low-temperature tolerance among different maize inbred lines. Therefore, future studies on identifying genes for low-temperature tolerance and improving maize varieties should focus more on phenotypic evaluations under artificial controlled low-temperature conditions.

Using the indoor relative germination rates in 2018 and 2019, four SNP markers significantly associated with low-temperature tolerance were identified in the range of 31,809,859–31,954,983 on chromosome 1, with an average distance of 36.28 Kb between markers. On chromosome 3, two SNP markers significantly associated with low-temperature tolerance were identified, namely marker.8339 and marker.8340. These markers can be directly applied or developed into easily detectable molecular markers for use in the marker-assisted selection for low-temperature tolerance in maize. In addition, some markers were found to be significantly associated with multiple traits, such as marker.1723 and marker.1724, which are only 43 bp apart and were associated with both indoor germination rates in 2018 and 2019. Marker.8339 and marker.8340 were associated with both indoor relative germination rates in 2018 and 2019, and marker.14070 was associated with both the field average relative germination rate and the 2019 indoor relative germination rate. The reason for the same markers being associated with multiple traits may be due to the strong correlation between traits, and it also indicates that the traits identified in this study are all effective for low temperature-tolerance identification. Another reason may be due to the pleiotropy of genes, where genes not only directly control the expression of a trait through the action of enzymes, but also affect many other traits through the modification of a particular trait. This requires further investigation into the function of candidate genes to avoid any negative effects of gene expression on maize breeding.

## 5. Conclusions

In the present study, GWAS was performed with 296 maize inbred lines, and a total of 14 SNPs significantly associated with low-temperature tolerance were detected. The SNP consistently linked to low-temperature tolerance in the field and indoors during germination was marker.14070, located on chromosome 5 at position 2,205,723, which explained 4.84–9.68% of the phenotypic variation.

## Figures and Tables

**Table 1:** The ANOVA analysis of traits related to low-temperature tolerance in the field from germination to the seedling stage.

Traits	Source of Variation	Degrees of Freedom	Sum of Squares	Mean Squares	*F*-Value	*p*-Value
	error	565	72,604	129	-	-
Relative germination index	Genotype	292	191,859	657	87.98	<2 × 10^−10^ ***
	Environment	1	4243	4243	568.15	<2 × 10^−10^ ***
	Block	1	4	4	0.52	0.47
	Genotype × environment	281	195,408	695	93.12	<2 × 10^−10^ ***
	Error	574	4287	7	-	-
Relative germination rate	Genotype	293	113,856	389	1.01	0.47
	Environment	1	12,858	12,858	33.33	<2 × 10^−8^ ***
	Error	285	109,946	386	-	-

Note: ‘***’ indicates significance at the 0.001 level.

**Table 2:** The low-temperature tolerance phenotype statistics from germination to the seedling stage.

Traits	Number	Min.	Max.	Mean	Median	SD	Kurtosis	Skewness
Relative germination rate	293	28.00	100.00	66.62	67.00	14.10	−0.39	−0.25
Relative germination index in 2017	291	20.69	100.00	86.80	92.68	16.65	2.78	−1.66
Relative germination index in 2018	284	4.08	100.00	83.15	89.70	20.02	0.85	−1.17

**Table 3:** The ANOVA analysis of traits related to low-temperature tolerance in the laboratory during the germination stage.

Traits	Source of Variation	Degrees of Freedom	Sum of Squares	Mean Squares	*F*-Value	*p*-Value
Relative germination rate	Genotype	287	478,817	1668	12.98	<2 × 10^−10^ ***
	Environment	1	70,893	70,893	551.68	<2 × 10^−10^ ***
	Block	1	2	2	0.012	0.91
	Genotype × environment	280	98,028	350	2.72	<2 × 10^−10^ ***
	Error	565	72,604	129	-	-

Note: ‘***’ indicates significance at the 0.001 level.

**Table 4:** The low-temperature tolerance phenotype statistics during the germination stage.

Traits	Number	Min	Max	Mean	Median	SD	Kurtosis	Skewness
Relative germination index in 2018	281	0.00	100.00	79.51	76.00	25.25	0.09	−0.10
Relative germination index in 2019	288	0.00	100.00	84.60	92.00	20.09	4.68	−2.12

**Table 5:** The correlation analysis of low-temperature tolerance during the germination stage.

Traits	Correlation Coefficient				
	Indoor Relative Germination Rate (2018)	Indoor Relative Germination Rate (2019)	Field Relative Germination Rate	Field Relative Germination Index (2017)	Field Relative Germination Index (2018)
Indoor relative germination rate (2018)	1	0.67 ***	0.18 ***	0.07	0.02
Indoor relative germination rate (2019)	0.67 ***	1	0.20 ***	0.02	0.11
Field-relative germination rate	0.18 ***	0.20 ***	1	0.50 ***	0.49 ***
Field-relative germination index (2017)	0.07	0.02	0.50 ***	1	−0.06
Field-relative germination index (2018)	0.02	0.11	0.49 ***	−0.06	1

Note: ‘***’ indicates significance at the 0.001 level.

**Table 6:** The allele frequency characteristics of SNP markers.

Chr.	SNP	Minor Allele Frequency (MAF)					
		>0.05	Percent (%)	>0.1	Percent (%)	>0.2	Percent (%)
1	3687	3599	98	1383	38	115	3
2	3217	3159	98	1174	36	89	3
3	2896	2833	98	930	32	91	3
4	2882	2802	97	947	33	95	3
5	2384	2334	98	838	35	94	4
6	1898	1868	98	715	38	90	5
7	1577	1532	97	580	37	57	4
8	1848	1793	97	635	34	62	3
9	2294	2264	99	854	37	111	5
10	1359	1313	97	529	39	36	3
Total	24,042	23,497	98	8585	36	840	3

**Table 7:** LD and LD attenuation of maize chromosomes.

Chr.	LD Decay (*r*^2^ < 0.2)	LD Decay (*r*^2^ < 0.1)	Min	Max	Mean	Median	SD	Kurtosis	Skewness
1	120	410	0.000	1.000	0.062	0.008	0.152	13.871	3.605
2	320	1000	0.000	1.000	0.088	0.009	0.201	6.198	2.69
3	130	620	0.000	1.000	0.067	0.007	0.169	10.411	3.264
4	100	500	0.000	1.000	0.061	0.008	0.152	13.999	3.648
5	270	990	0.000	1.000	0.082	0.008	0.187	7.887	2.903
6	85	350	0.000	1.000	0.064	0.008	0.16	11.833	3.435
7	90	440	0.000	1.000	0.052	0.008	0.141	17.75	4.069
8	220	820	0.000	1.000	0.068	0.008	0.169	11.335	3.378
9	340	1990	0.000	1.000	0.122	0.011	0.24	2.903	2.069
10	70	340	0.000	1.000	0.07	0.01	0.165	11.326	3.351

Note: chr.1 to chr.10 represent chromosome 1 to chromosome 10 in maize, respectively.

**Table 8:** SNPs of maize with significant correlation with low-temperature tolerance.

Traits	Marker	Chr.	Physical Position	−*Lg* (*p*)	Contribution (%)
Field-relative germination rate	marker.14070	5	2,205,723	6.86	9.68
Relative germination index (2018)	marker.17002	6	64,236,775	4.56	6.55
Relative germination index (2018)	marker.17003	6	64,236,781	4.56	6.55
Relative germination index (2018)	marker.17009	6	64,298,566	4.15	5.86
Relative germination index (2018)	marker.17105	6	72,142,751	5.69	8.46
Relative germination index (2018)	marker.19874	7	180,326,388	5.01	7.30
Field-relative germination rate	marker.536	10	63,529,769	4.06	5.03

Note: The physical position of SNP markers was determined in reference to B73 RefGen-v4.

**Table 9:** SNPs of maize with significant correlations with low-temperature tolerance.

Traits	Marker	Chr.	Physical Position	−*Lg* (*p*)	Contribution (%)
Relative germination rate (2019)	marker.1723	1	31,809,859	5.01	6.54
Relative germination rate (2018)	marker.1724	1	31,809,902	4.07	5.46
Relative germination rate (2019)	marker.1726	1	31,897,277	4.18	5.31
Relative germination rate (2019)	marker.1729	1	31,954,983	4.95	6.41
Relative germination rate (2018)	marker.8339	3	6,292,001	4.16	5.61
Relative germination rate (2019)	marker.8339	3	6,292,001	5.42	7.12
Relative germination rate (2018)	marker.8340	3	6,292,053	5.01	6.87
Relative germination rate (2019)	marker.8340	3	6,292,053	4.87	6.34
Relative germination rate (2019)	marker.12816	4	140,575,088	4.54	5.87
Relative germination rate (2018)	marker.14070	5	2,205,723	5.17	6.26
Relative germination rate (2018)	marker.190	10	22,696,941	4.13	5.56
Relative germination rate (2019)	marker.753	10	90,874,322	4.75	6.17
Relative germination rate (2019)	marker.14070	5	2,205,723	4.00	4.84
Relative germination rate (2018)	marker.843	10	100,622,715	4.52	6.10

Note: The physical position of SNP markers was determined in reference to B73 RefGen-v4.

## Data Availability

The data used in this study are available within the text.

## Appendix A

**Table A1:** The population distribution characteristics when the number of subgroups is 5.

NO.	Name	Ancestry Matrix					Subgroup
		A	B	C	D	E	
1	LX46	0.86	0	0.05	0	0.09	A
2	UH302	0.92	0	0	0.08	0	A
3	LX56	0.57	0.14	0	0	0.29	A
4	LX57	0.59	0.14	0	0	0.27	A
5	LX65	0.52	0.02	0.37	0.05	0.04	A
6	LX76	0.39	0.09	0.32	0	0.2	A
7	LX98	0.9	0.1	0	0	0	A
8	LX99	0.67	0.33	0	0	0	A
9	38P05f	0.77	0	0.07	0	0.16	A
10	38P05m	1	0	0	0	0	A
11	LX113	1	0	0	0	0	A
12	YA1M	0.9	0	0.04	0.03	0.03	A
13	LX147	0.85	0	0.06	0.03	0.06	A
14	LX148	0.88	0	0.05	0.03	0.04	A
15	LX151	0.74	0.09	0	0	0.17	A
16	LX156	0.67	0.33	0	0	0	A
17	LX162	0.37	0	0.37	0.16	0.1	A
18	LX171	0.53	0.03	0.06	0.2	0.18	A
19	UH303	0.91	0	0.05	0.04	0	A
20	LX194	0.45	0.15	0.27	0	0.13	A
21	688F	0.94	0.06	0	0	0	A
22	LX94	0	0.48	0.23	0.01	0.28	B
23	LX115	0.01	0.56	0.02	0	0.41	B
24	xy335f	0	0.97	0.03	0	0	B
25	LX132	0.01	0.44	0.15	0	0.4	B
26	LX137	0.04	0.47	0.11	0	0.38	B
27	LY88M	0	0.78	0.06	0	0.16	B
28	LX164	0	0.65	0.07	0	0.28	B
29	LX166	0	0.68	0	0	0.32	B
30	LX167	0	0.68	0.04	0.02	0.26	B
31	LX168	0	0.54	0.01	0	0.45	B
32	LX169	0	0.61	0.05	0	0.34	B
33	LX172	0.03	0.55	0	0	0.42	B
34	LX180	0	0.88	0.1	0.02	0	B
35	LX186	0	0.89	0.09	0.02	0	B
36	LX205	0	0.51	0.26	0	0.23	B
37	LX206	0.04	0.48	0.24	0.03	0.21	B
38	LY99M	0	1	0	0	0	B
39	MEIXI	0.03	0.43	0.17	0.04	0.33	B
40	101M	0.01	0.53	0	0	0.46	B
41	420F	0	1	0	0	0	B
42	738M	0	1	0	0	0	B
43	820F	0.05	0.55	0.33	0.07	0	B
44	LX1	0.05	0.04	0.49	0.09	0.33	C
45	LX2	0.03	0.05	0.62	0.16	0.14	C
46	LX3	0.01	0.06	0.64	0.2	0.09	C
47	LX4	0	0.13	0.53	0.17	0.17	C
48	LX5	0.02	0.02	0.83	0.13	0	C
49	LX6	0	0.02	0.92	0.04	0.02	C
50	LX8	0.02	0.02	0.59	0.04	0.33	C
51	LX9	0	0	1	0	0	C
52	LX10	0.03	0.03	0.76	0.05	0.13	C
53	LuYuan92	0.02	0.03	0.72	0.08	0.15	C
54	LX12	0.25	0	0.45	0.22	0.08	C
55	LX13	0.02	0.05	0.53	0.05	0.35	C
56	LX14	0.02	0.05	0.59	0.04	0.3	C
57	LX15	0.03	0.09	0.69	0.05	0.14	C
58	LX17	0.02	0.03	0.69	0.04	0.22	C
59	LX18	0.1	0.03	0.44	0.3	0.13	C
60	H261	0	0.02	0.74	0.05	0.19	C
61	LX19	0.05	0.05	0.61	0.05	0.24	C
62	LX20	0	0.01	0.61	0.05	0.33	C
63	LX21	0.19	0.08	0.37	0.07	0.29	C
64	Q319	0.04	0.06	0.71	0.05	0.14	C
65	LX23	0.02	0.05	0.73	0.18	0.02	C
66	LX25	0.1	0.08	0.6	0.07	0.15	C
67	LX95	0	0.05	0.74	0.21	0	C
68	LX27	0	0	0.86	0	0.14	C
69	LX29	0.04	0.04	0.66	0.07	0.19	C
70	LX30	0	0.08	0.56	0.29	0.07	C
71	LX32	0	0	0.88	0.12	0	C
72	LX33	0	0.07	0.57	0.31	0.05	C
73	LX34	0	0.02	0.75	0.16	0.07	C
74	LX35	0.1	0.05	0.45	0.26	0.14	C
75	LX36	0.27	0.01	0.4	0.21	0.11	C
76	LX37	0.04	0.06	0.46	0.28	0.16	C
77	LX38	0	0	0.97	0	0.03	C
78	LX39	0	0	0.82	0.18	0	C
79	LX40	0.03	0	0.71	0	0.26	C
80	LX41	0	0	0.54	0.15	0.31	C
81	LX42	0.01	0.05	0.47	0.03	0.44	C
82	LX43	0	0	0.87	0.13	0	C
83	LX45	0.02	0.03	0.78	0.08	0.09	C
84	B317	0	0.01	0.84	0.08	0.07	C
85	LX48	0.01	0.02	0.77	0.11	0.09	C
86	B144	0.02	0.04	0.47	0.04	0.43	C
87	Si287	0	0	0.84	0.16	0	C
88	7-004	0	0	0.66	0	0.34	C
89	SD190	0.01	0.05	0.78	0.08	0.08	C
90	LX49	0.02	0.03	0.73	0.01	0.21	C
91	LX50	0	0	0.89	0	0.11	C
92	LX52	0.05	0	0.69	0.05	0.21	C
93	LX53	0	0	0.9	0.05	0.05	C
94	LX55	0.01	0.05	0.62	0.16	0.16	C
95	LX59	0.06	0.02	0.72	0.05	0.15	C
96	LX60	0.02	0.04	0.62	0.08	0.24	C
97	LX61	0.03	0.03	0.73	0.12	0.09	C
98	LX62	0	0	0.76	0.24	0	C
99	LX63	0	0.08	0.79	0.1	0.03	C
100	LX64	0	0.01	0.75	0.2	0.04	C
101	LX66	0.23	0	0.42	0.13	0.22	C
102	LX67	0.05	0.07	0.53	0.08	0.27	C
103	LX68	0.02	0.04	0.33	0.29	0.32	C
104	LX69	0.02	0.01	0.55	0.29	0.13	C
105	LX71	0.02	0	0.87	0.04	0.07	C
106	LX74	0	0.21	0.58	0	0.21	C
107	LX75	0.04	0.18	0.41	0.05	0.32	C
108	LX82	0.03	0.01	0.48	0.22	0.26	C
109	LX83	0	0	0.74	0.22	0.04	C
110	LX84	0	0	0.8	0.19	0.01	C
111	LX85	0.03	0.06	0.49	0.02	0.4	C
112	LX86	0	0	0.93	0.07	0	C
113	LX87	0	0	0.95	0.05	0	C
114	LX90	0.02	0.04	0.47	0.06	0.41	C
115	LX92	0	0	0.74	0.26	0	C
116	LX93	0	0	0.66	0.3	0.04	C
117	LX100	0	0	0.53	0.29	0.18	C
118	LX102	0	0.03	0.86	0.09	0.02	C
119	LM33M	0.03	0.05	0.61	0.07	0.24	C
120	LX105	0.05	0	0.88	0.05	0.02	C
121	LX106	0.01	0.1	0.81	0.05	0.03	C
122	LX107	0.06	0	0.89	0.05	0	C
123	LX112	0.07	0.14	0.47	0.09	0.23	C
124	LX114	0.06	0.12	0.4	0.02	0.4	C
125	Huangzao4	0	0	0.86	0.14	0	C
126	Longxi53	0.04	0.04	0.41	0.13	0.38	C
127	706	0.08	0.05	0.71	0.11	0.05	C
128	LX117	0.01	0.01	0.96	0.02	0	C
129	LX118	0.03	0.1	0.72	0.06	0.09	C
130	LX119	0	0.03	0.78	0.14	0.05	C
131	K10	0	0.09	0.47	0.35	0.09	C
132	Chang3	0.02	0.03	0.6	0.13	0.22	C
133	Zhong7490-92	0.01	0.04	0.76	0.18	0.01	C
134	78599	0.03	0	0.91	0.06	0	C
135	478	0	0	0.89	0	0.11	C
136	He344	0	0.01	0.75	0.18	0.06	C
137	Longkang11	0	0	1	0	0	C
138	MO17	0.04	0.17	0.56	0.03	0.2	C
139	4F1	0	0	1	0	0	C
140	330	0	0	0.9	0.1	0	C
141	5003	0	0	0.98	0.02	0	C
142	B73	0.03	0.01	0.96	0	0	C
143	LX120	0.07	0.03	0.66	0.19	0.05	C
144	LX121	0.02	0.02	0.87	0.07	0.02	C
145	LX122	0	0.04	0.69	0.27	0	C
146	LX123	0.03	0	0.83	0.11	0.03	C
147	LX124	0	0	0.99	0.01	0	C
148	06S021	0	0	0.97	0	0.03	C
149	06S032	0	0	1	0	0	C
150	06S034	0.13	0.01	0.78	0.05	0.03	C
151	06S052	0	0	0.53	0.01	0.46	C
152	06S060	0	0.01	0.84	0	0.15	C
153	06S068	0	0	0.92	0	0.08	C
154	06S075	0	0	0.77	0.14	0.09	C
155	06S095	0	0.04	0.71	0.1	0.15	C
156	R117	0.01	0	0.91	0	0.08	C
157	FLAF	0.13	0.01	0.48	0.22	0.16	C
158	YAM	0.07	0.08	0.66	0.09	0.1	C
159	LX125	0	0.05	0.81	0.14	0	C
160	Jidan27♂	0.01	0.01	0.49	0.04	0.45	C
161	LX126	0	0	0.59	0.01	0.4	C
162	LX127	0	0.03	0.75	0.04	0.18	C
163	LX128	0	0.02	0.83	0.13	0.02	C
164	LX129	0	0	0.98	0.02	0	C
165	LX130	0.01	0	0.67	0.01	0.31	C
166	LX131	0.03	0.13	0.61	0.08	0.15	C
167	Zheng58	0.01	0	0.87	0	0.12	C
168	Chang7-2	0	0.01	0.8	0.14	0.05	C
169	HY6M	0.18	0	0.49	0.19	0.14	C
170	HY6F	0.04	0.03	0.77	0.06	0.1	C
171	LX134	0.26	0	0.64	0.08	0.02	C
172	LX140	0	0	0.87	0	0.13	C
173	LX144	0.03	0.09	0.51	0.06	0.31	C
174	Co117-2	0.1	0.04	0.53	0.19	0.14	C
175	Co220	0.09	0.02	0.47	0.3	0.12	C
176	Co228	0.15	0.03	0.4	0.3	0.12	C
177	Co266	0.09	0.04	0.44	0.31	0.12	C
178	Co274	0.07	0.09	0.55	0.15	0.14	C
179	Co285	0.07	0.02	0.51	0.08	0.32	C
180	Co358	0.13	0.02	0.48	0.21	0.16	C
181	Co372	0.07	0.02	0.49	0.27	0.15	C
182	Co373	0.1	0.06	0.47	0.28	0.09	C
183	Co380	0.08	0.03	0.46	0.19	0.24	C
184	LY88F	0	0	0.8	0.1	0.1	C
185	LX149	0	0	0.94	0.06	0	C
186	LX150	0	0	0.81	0.08	0.11	C
187	LX152	0.02	0	0.93	0	0.05	C
188	LX153	0.06	0.02	0.71	0.09	0.12	C
189	LX154	0.01	0	0.87	0.12	0	C
190	LX155	0	0.02	0.83	0.15	0	C
191	H127RE	0.04	0.04	0.64	0.12	0.16	C
192	LX163	0.05	0.07	0.82	0	0.06	C
193	DY1	0	0.01	0.88	0.11	0	C
194	DY10	0.01	0.03	0.53	0	0.43	C
195	DY21	0.07	0.12	0.44	0.03	0.34	C
196	DY53	0.01	0	0.86	0.13	0	C
197	DY71	0.02	0.02	0.8	0.16	0	C
198	DY97	0	0.01	0.63	0	0.36	C
199	DY99	0.03	0.03	0.5	0.22	0.22	C
200	Dy13-17	0.14	0.03	0.46	0.2	0.17	C
201	LX176	0.03	0.02	0.95	0	0	C
202	LX177	0	0.06	0.9	0.04	0	C
203	LX178	0.03	0.06	0.57	0.06	0.28	C
204	LX181	0	0.07	0.59	0.06	0.28	C
205	LX184	0.01	0.03	0.67	0.01	0.28	C
206	LX185	0.04	0.22	0.48	0.04	0.22	C
207	LX188	0.03	0.26	0.37	0.02	0.32	C
208	LX190	0.09	0.17	0.4	0.02	0.32	C
209	LX193	0	0.02	0.95	0.03	0	C
210	LX195	0	0.01	0.91	0.08	0	C
211	LX196	0.07	0.01	0.77	0.03	0.12	C
212	LX197	0	0.01	0.75	0.01	0.23	C
213	LX198	0	0.01	0.77	0.01	0.21	C
214	LX199	0.02	0	0.76	0	0.22	C
215	LX201	0.04	0.02	0.49	0.02	0.43	C
216	LX202	0.05	0	0.44	0.08	0.43	C
217	LX209	0.13	0.06	0.67	0.08	0.06	C
218	LX210	0.13	0.13	0.54	0.06	0.14	C
219	698F	0.04	0.04	0.68	0.01	0.23	C
220	820M	0.13	0.14	0.37	0.05	0.31	C
221	YA2M	0.27	0.09	0.47	0.06	0.11	C
222	LX26	0	0.03	0.17	0.69	0.11	D
223	UH004	0	0	0	1	0	D
224	FLAM	0	0.04	0.24	0.58	0.14	D
225	YAF	0	0	0	0.97	0.03	D
226	Co374	0.21	0.03	0.2	0.45	0.11	D
227	YA1F	0	0.02	0	0.94	0.04	D
228	LX159	0.36	0	0	0.64	0	D
229	LX160	0.47	0	0	0.53	0	D
230	LX161	0.21	0.1	0.06	0.52	0.11	D
231	UH009	0	0.01	0	0.93	0.06	D
232	Ji871	0	0	0.39	0.1	0.51	E
233	LX11	0.04	0.03	0.42	0.08	0.43	E
234	LX16	0	0.27	0.25	0.02	0.46	E
235	LX22	0	0	0.12	0.03	0.85	E
236	LX24	0	0.22	0	0	0.78	E
237	LX31	0.03	0.03	0.41	0.04	0.49	E
238	LX51	0.01	0	0.36	0.05	0.58	E
239	LX54	0	0	0.21	0	0.79	E
240	LX72	0.34	0.03	0.09	0	0.54	E
241	LX73	0.05	0.26	0.25	0.07	0.37	E
242	LX77	0.16	0.11	0.26	0.03	0.44	E
243	LX78	0.14	0.18	0.06	0	0.62	E
244	LX79	0.15	0.19	0.04	0	0.62	E
245	LX80	0.06	0.11	0.21	0.01	0.61	E
246	LX88	0.19	0.11	0.28	0	0.42	E
247	LX89	0.03	0.02	0.34	0.02	0.59	E
248	LX91	0	0	0.09	0.11	0.8	E
249	LX101	0.06	0.25	0.27	0.04	0.38	E
250	LX104	0	0	0.13	0.01	0.86	E
251	LX108	0	0.1	0	0	0.9	E
252	LX109	0.02	0.17	0.24	0.02	0.55	E
253	LX110	0.13	0.01	0.03	0.04	0.79	E
254	LX111	0.02	0.05	0.33	0.05	0.55	E
255	LX116	0	0.02	0.01	0	0.97	E
256	Lv28	0	0	0	0	1	E
257	LX124	0.38	0.01	0.21	0	0.4	E
258	xy335m	0	0.09	0.02	0	0.89	E
259	LX133	0	0.41	0.16	0.01	0.42	E
260	LX135	0	0.33	0.09	0.01	0.57	E
261	LX136	0	0.43	0.12	0.01	0.44	E
262	LX138	0	0.34	0.23	0.02	0.41	E
263	LX139	0.06	0.23	0.23	0.03	0.45	E
264	LX141	0.02	0.08	0.37	0.06	0.47	E
265	LX142	0	0	0	0	1	E
266	LX143	0	0	0	0	1	E
267	LX145	0	0	0	0.02	0.98	E
268	LX146	0.01	0.02	0.14	0.01	0.82	E
269	Co371	0.07	0.09	0.28	0.17	0.39	E
270	LX165	0.3	0.04	0.3	0.01	0.35	E
271	LX170	0.02	0.41	0	0	0.57	E
272	DY7	0	0	0	0	1	E
273	DY29	0.01	0	0	0.01	0.98	E
274	DY36	0.05	0.03	0.14	0.02	0.76	E
275	DY49	0	0.12	0.2	0	0.68	E
276	LX173	0	0.07	0.09	0.02	0.82	E
277	LX174	0	0	0	0	1	E
278	LX175	0	0	0.04	0	0.96	E
279	LX179	0	0.09	0.34	0.05	0.52	E
280	LX182	0.42	0.09	0	0.02	0.47	E
281	LX183	0.06	0.1	0.24	0.2	0.4	E
282	LX187	0.01	0.03	0.36	0.07	0.53	E
283	LX191	0.04	0.23	0.27	0.04	0.42	E
284	LX192	0.18	0.26	0.24	0.01	0.31	E
285	LX200	0	0	0.45	0.04	0.51	E
286	LX203	0	0	0	0	1	E
287	LX204	0	0	0	0	1	E
288	LX207	0	0	0	0	1	E
289	LX208	0	0.07	0.18	0.02	0.73	E
290	252M	0.01	0.06	0.21	0.01	0.71	E
291	335MG	0	0.04	0	0	0.96	E
292	688M	0	0.04	0	0	0.96	E
293	XZD276	0	0.1	0	0	0.9	E
294	XZD170	0	0.08	0	0	0.92	E
295	XZD171	0.02	0.13	0.31	0.02	0.52	E
296	YA2F	0	0.33	0	0	0.67	E
